# Editorial: Impact of new vitamin D guidelines on pediatric and adult health: insights, evidence, and implications

**DOI:** 10.3389/fendo.2026.1851754

**Published:** 2026-04-29

**Authors:** Benjamin Udoka Nwosu, Cristina Vassalle

**Affiliations:** 1 Donald and Barbara Zucker School of Medicine, Hofstra University, Hempstead, NY, United States; 2 Department of Pediatrics, Fondazione Toscana Gabriele Monasterio per la Ricerca Medica e di Sanita Pubblica, Pisa, Italy

**Keywords:** cholecalciferol (vitamin D3), ergocalciferol (vitamin D2), guideline, infants, pregnancy, vitamin D, vitamin D deficiency

The 2024 Endocrine Society Clinical Practice Guideline (1) on Vitamin D for the Prevention of Disease remains controversial (1, 2). This one-size-fits-all recommendation largely ignored the realities of current research in vitamin D metabolism where the extra-skeletal functions of vitamin D are being established by well-designed clinical studies (3–6). While the 2024 Guideline focuses on standardizing recommendations for vitamin D intake and supplementation, it downplayed the impact of such recommendations across diverse populations such as fetuses, children, pregnant women, obese- and dark-skinned individuals.

This Research Topic explores the evidence-based and real-world impact of the 2024 Vitamin D Guideline from multiple perspectives, highlighting the Guideline’s strengths and weaknesses, and identifying areas for future research.

In the section on the metabolic consequences of undetected vitamin D deficiency, Nguyen et al. from Vietnam examined the etiology and consequences of severe pediatric hypocalcemia in a tropical country. They studied 246 children and adolescents, age 0–18 years (median age, 67 days) who were admitted with severe hypocalcemia to a tertiary institution in Vietnam between 2018-2024. This report cuts through the one-size-fits-all 2024 Endocrine Society Guideline (1) to show that severe hypocalcemia in this tropical country was primarily due to preventable vitamin D deficiency (67.1% of cases), and severe hypoparathyroidism (28% of cases). The consequences of the severe hypocalcemia included seizures in 79.2% of the children, and cardiac complications such as cardiogenic shock and acute heart failure in 5.7% of cases. This clinical scenario in a tropical country with ample sunshine, where vitamin D deficiency should be rare, shows the limitations of the 2024 Guideline (1) which did not stipulate routine vitamin D screening, monitoring, and treatment of vitamin D deficiency in infants and children.

In the section on the controversies on vitamin D status designation, Zhu et al. showed that the disparate cut-off values or thresholds for vitamin D status by different medical scientific societies might be harmful to patients, as deficiency and sufficiency states easily become a game of numbers, to the detriment of untreated patients who may fall into the wrong designation. This report assessed the vitamin D status of 1183 healthy children, aged 0–6 years in China, from 2022-2023. It found that different diagnostic thresholds for vitamin D status alter the classification of deficiency/sufficiency from 2.46% to 12.3%. As a result, this paper recommends a pragmatic approach to vitamin D status designation, by calling for region-specific guidelines that are based on latitudes; and for targeted vitamin D supplementation protocols that account for seasonal differences in vitamin D status, such as increased supplementation protocol in winter. This reasoned approach contrasts with the monolithic recommendation from the Endocrine Society.

In the section on asthma, Dong et al. reported a high prevalence of vitamin D deficiency of 45.2%, and insufficiency of 31.0%, in 320 school-age children in China. They also showed that vitamin D deficiency is a major predictor of asthma morbidity after adjusting for confounders.

In diabetology, the 2024 Guideline (1) made no provision for vitamin D supplementation to prevent the complications of type 1 diabetes (T1D) or type 2 diabetes (T2D). To examine this exclusion, Jiang et al. conducted a 3-day functional study in 911 adult patients with T2D that included continuous glucose monitoring and mixed meal tolerance test. They reported that higher time in range and time in tight range occurred in patients in the upper tertiles for serum 25-hydroxyvitamin D [25(OH)D]. They also found that serum 25(OH)D correlated positively with the markers of early phase and overall pancreatic β-cell secretory capacity. This is consistent with the finding of β-cell protection by high-dose vitamin D supplementation in children with T1D (6). In a case report, Chader-Gata Garcia et al. showed that high-dose, adjunctive vitamin D supplementation could improve glycemia and prolong the partial clinical remission phase of T1D in a child for more than 27 months.

In the section on cancer pathogenesis, Hendi et al. explored a possible genetic basis for vitamin D deficiency. They found that the deficiency of *SDR42E1* gene, a key modulator of vitamin D related pathways, reduced cell viability by 53% (p=0.0001); and that this reduction in cell viability could be reversed by transient *SDR42E1* overexpression to restore ABCC2 expression. They suggested that this could serve as a therapeutic target to restore vitamin D sufficiency and prevent the associated deleterious effects, especially in cancers. In a meta-analysis of 23 cross-sectional studies, Yang et al. found a significant association between vitamin D deficiency and increased risk of thyroid cancer. This is important as there is an upregulation of vitamin D receptor genes and other genes associated with vitamin D metabolism in malignant thyrocytes (7). The authors called for well-designed prospective studies to explore their findings.

In the section on cardiology, Ilmer et al. report on the resolution of complete heart block (CHB) in a child with Graves’ disease following the institution of adjunctive high-dose vitamin D supplementation. This report on CHB is supported by earlier findings on the efficacy of high-dose vitamin D on non-skeletal health (3–6, 8).

In a rebuttal to the 2024 Guideline (1), Nwosu rejects the sweeping one-size-fits-all recommendations, and the opaque recommendations for infants, children, adolescents, obese individuals, darkly pigmented individuals, pregnant women and fetuses. This rebuttal calls for routine screening, supplementation, and monitoring of serum 25(OH)D concentrations to ensure that everyone benefits from the skeletal and extra-skeletal actions of vitamin D.

In conclusion, this Research Topic employed evidence-based and real-world research to examine the 2024 Endocrine Society Guideline on Vitamin D for Prevention of Disease by focusing on the role of vitamin D deficiency on childhood hypocalcemia; the health impact of disparate cut-off points for vitamin D status designation by different medical societies; the impact of high-dose vitamin D supplementation on T1D, T2D, and complete heart block; the negative impact of vitamin D deficiency in asthma, thyroid cancer, and diabetes mellitus. There is also the exciting possibility that transient overexpression of *SDR42E1* gene can reverse these vitamin D deficiency states. These innovative works will spur further research in this field, and will shape future recommendations for vitamin D screening, monitoring, and supplementation.
